# Protective Mechanisms of Avocado Oil Extract Against Ototoxicity

**DOI:** 10.3390/nu12040947

**Published:** 2020-03-29

**Authors:** Thu Nguyen Minh Pham, Seo Yeon Jeong, Do Hoon Kim, Yu Hwa Park, Jung Suk Lee, Kye Wan Lee, In Seok Moon, Se Young Choung, Seung Hyun Kim, Tong Ho Kang, Kwang Won Jeong

**Affiliations:** 1 Gachon Research Institute of Pharmaceutical Sciences, College of Pharmacy, Gachon University, 191 Hambakmoero, Yeonsu-gu, Incheon 21936, Korea; phamnguyenminhthud12@gmail.com (T.N.M.P.); syjeong46@naver.com (S.Y.J.); 2 R&D Center, Dongkook Pharm. Co., Ltd., Gyeonggi 16229, Korea; Kdh2@dkpharm.co.kr (D.H.K.); pyh@dkpharm.co.kr (Y.H.P.); ljs@dkpharm.co.kr (J.S.L.); lkw1@dkpharm.co.kr (K.W.L.); 3 Department of Otorhinolaryngology, Yonsei University College of Medicine, Seoul 03722, Korea; ISMOONMD@yuhs.ac; 4 Department of Life and Nanopharmaceutical Sciences, College of Pharmacy, Graduate School, Kyung Hee University, Dongdaemun-gu, Seoul 02447, Korea; sychoung@khu.ac.kr; 5 College of Pharmacy, Yonsei Institute of Pharmaceutical Sciences, Yonsei University, Incheon 21983, Korea; kimsh11@yonsei.ac.kr; 6 Department of Oriental Medicine Biotechnology, College of Life Sciences and Graduate School of Biotechnology, Kyung Hee University, Global Campus, Gyeonggi 17104, Korea

**Keywords:** avocado oil, aminoglycoside, ototoxicity, hearing loss, ear hair cell

## Abstract

Despite the excellent antimicrobial activity of aminoglycoside antibiotics, permanent inner ear damage associated with the use of these drugs has resulted in the need to develop strategies to address the ototoxic risk given their widespread use. In a previous study, we showed that avocado oil protects ear hair cells from damage caused by neomycin. However, the detailed mechanism by which this protection occurs is still unclear. Here, we investigated the auditory cell-protective mechanism of enhanced functional avocado oil extract (DKB122). RNA sequencing followed by pathway analysis revealed that DKB122 has the potential to enhance the expression of detoxification and antioxidant genes associated with glutathione metabolism (Hmox4, Gsta4, Mgst1, and Abcc3) in HEI-OC1 cells. Additionally, DKB122 effectively decreased ROS levels, resulting in the inhibition of apoptosis in HEI-OC1 cells. The expression of the inflammatory genes that encode chemokines and interleukins was also downregulated by DKB122 treatment. Consistent with these results, DKB122 significantly inhibited p65 nuclear migration induced by TNF-α or LPS in HEI-OC1 cells and THP-1 cells and the expression of inflammatory chemokine and interleukin genes induced by TNF-α was significantly reduced. Moreover, DKB122 treatment increased LC3-II and decreased p62 in HEI-OC1 cells, suggesting that DKB122 increases autophagic flux. These results suggest that DKB122 has otoprotective effects attributable to its antioxidant activity, induction of antioxidant gene expression, anti-inflammatory activity, and autophagy activation.

## 1. Introduction

Approximately 466 million people around the world experience hearing loss, including 34 million children [1]. Aside from congenital causes, the etiologies of hearing loss include ear infections, noise, aging, and ototoxic chemicals. It is believed that excessive noise and ototoxic chemicals result in inflammation of the inner ear, and drug ototoxicity, in particular, is one of the most preventable contributors to hearing loss. Aminoglycosides (AGs) are a well-known class of antibiotics for use against infections caused by gram-negative bacteria. AGs are both nephrotoxic [2] and ototoxic [3]. Despite their deleterious effects on the nephron and inner ear, AGs remain the most widely used antibiotics. Among AGs, neomycin is characterized as the most toxic, specifically in the cochlea [4].

AGs are known for harming hair cells in the cochlea. At basal rotation, outer hair cells are initially damaged. The AG then damages the inner hair cells, stria vascularis, the marginal cells, and the spiral ganglion [5]. Previous work has demonstrated the mechanism by which AGs form complexes with ferric cations in the cytoplasm to generate reactive oxygen species (ROS) using arachidonic acid as the electron donor. Excessive ROS causes cell death through various mechanisms, including apoptosis and necrosis [6]. Other studies have proposed that AGs deplete free phosphoinositides that regulate KCNQ4 channel activity, thereby inhibiting the potassium outflow required for cochlear sensory function and outer hair cell survival [7].

The House Ear Institute-Organ of Corti 1 (HEI-OC1) cells are well characterized as an in vitro model to study ototoxicity-related effects and the molecular mechanism of ototoxicity [8,9]. For example, HEI-OC1 cells are susceptible to neomycin which increases ROS formation and eventually leads to cell death [9]. Consequently, ROS neutralization has become a potential therapeutic target for ototoxicity, as there are several antioxidants that have been investigated to attenuate the damage of ototoxic drugs such as acetylsalicylate, N-acetylcysteine, D-methionine, alpha-lipoic acid, and N-acetylcysteine [9,10]. In a previous study, we showed that avocado (*Persea americana Mill.*) oil extract protects functional ear cells from damage caused by neomycin [11]. Avocado contains a significant amount of oil, and avocado pulp is enriched with secondary metabolites such as carotenoids and tocopherols [12]. The composition of the common fatty acids of the oils varies according to avocado quality (varieties and geographical origin) and oil extraction methods. However, it is well recognized that oleic acid has a higher proportion than other individual fatty acids, followed by palmitic, linoleic, palmitoleic, and α-linolenic. Due to the differences in fatty acid and triacylglycerol composition, iodine value, slip melting point, melting, and solidification characteristics are completely different in different avocado varieties [13,14,15]. The health benefits of avocado oil are attributed to its high monounsaturated fatty acid content. Indeed, several bioactive compounds isolated from avocado have shown positive effects on human breast cancer and prostate cancer cells, along with hepatoprotective effects [16]. In addition, a dietary supplement of 5–20% avocado oil has been shown to reduce glucose tolerance and insulin resistance stimulated by a high-sucrose diet in Wistar rats [17]. Fatty acids in avocado oil can enhance collagen synthesis and reduce inflammation, accelerating wound healing [18]. The seed oil, in particular, significantly attenuates oxidative stress and prevents mitochondrial dysfunction in the brain tissue of diabetic rats [19]. Although the effect of avocado oil on auditory hair cells has been confirmed recently, the mechanism of action is yet to be determined.

Here, we investigated the mechanism of the avocado oil extract DKB122 on neomycin-induced ototoxicity in HEI-OC1 cells. We first confirmed the otoprotective activity in vitro by measuring cell viability and apoptosis. RNA-seq and pathway analysis were performed to shed light on the molecular mechanism of DKB122 on HEI-OC1 cells. In addition, we investigated the efficacy of DKB122 on ROS formation and inflammation, which are highly related to the ototoxicity induced by neomycin. Finally, we investigated the effect of DKB122 on autophagy in HEI-OC1 cells.

## 2. Materials and Methods

### 2.1. Sample Preparation

DKB122 (Dongkook Pharm. Co. ltd, Sowon, Korea, Lot number is BDP180313-3) was prepared as described previously [11]. Briefly, the DKB122, which is from crude avocado oil, was manufactured through ion exchange resin (Diaion HP-20, Mitsubishi Chemical Corp., Tokyo, Japan) with isopropyl alcohol. The preparation of the column was performed by soaking resin in 50% ethanol, and then packing it into a 3.5 cm diameter column up to 25 cm high. Avocado crude oil was dissolved in 50% ethanol, then loaded into the column, until the oil was absorbed into resin. Then, it was successively partitioned with water, 50% ethanol, and isopropyl alcohol. More than 56% of DKB122 is composed of four main fatty acids (palmitoleic acid, linoleic acid, palmitic acid, and oleic acid).

### 2.2. Cell Culture

House Ear Institute-Organ of Corti 1 (HEI-OC1) cells (House Ear Institute, Los Angeles, CA, USA) were cultured in Dulbecco’s modified Eagle’s medium containing 10% fetal bovine serum and 50 U/mL interferon-gamma (PEPROTECH, Rocky Hill, NJ, USA) under permissive condition (33 °C, 10% CO_2_) without antibiotics as previously described [11]. THP-1 human monocytic cell line was obtained from the Korean Cell Line Bank and maintained in RPMI 1640 medium supplemented with 10% FBS, 300 mg/L L-glutamine, 25 mM HEPES, and 25 mM NaHCO_3_ according to the standard protocol under normal conditions (37 °C, 5% CO_2_). For cell-based assays, DBK122 was used at a concentration of 1 to 100 μg/mL, which shows no direct cytotoxicity (Supplementary Figure S1A).

### 2.3. Cell Viability Assay

HEI-OC1 cells were plated at a density of 4 × 10^4^ cells/well in a 24-well plate. Cells were pretreated with DKB122 1 h before neomycin (10 mM) or cisplatin (30 μM) treatment. After 24 h incubation, cell viability was determined by IncuCyte^®^ (Panasonic, Tokyo, Japan).

### 2.4. mRNA Sequencing and Pathway Analysis

HEI-OC1 cells were plated at a density of 10^5^ cells/well in a 6-well plate. Cells were treated with 25 μg/mL DKB122 for 24 h. Total RNA was isolated using TRIzol RNA Isolation Reagents (Invitrogen, Carlsbad, CA, USA) and then further purified using the RNeasy mini kit (QIAGEN, Hilden, Germany). RNA-seq was performed as previously described [20]. Briefly, purified RNA was evaluated using the Agilent 2100 bioanalyzer RNA kit (Agilent, Santa Clara, CA, USA) and processed to prepare an mRNA-seq library using a TruSeq Stranded mRNA kit (Illumina, San Diego, CA, USA). The quality and size of libraries were assessed using the Agilent 2100 bioanalyzer DNA kit (Agilent). All libraries were quantified by qPCR using the CFX96 Real Time System (Bio-Rad, Hercules, CA, USA) and sequenced on a NextSeq500 sequencer (Agilent) with a paired-end 75 bp plus single 8 bp index read run. The raw data were converted into sequence data and stored in the FASTQ format. Genes showing absolute fold changes (FC) of at least 1.4 and *p* < 0.05 between groups were considered differentially expressed. The significantly differential expression datasets were approached using the KEGG expression database; WikiPathways analysis was performed using EnrichR [21].

### 2.5. Real Time-qPCR

Total RNA was isolated from ARPE-19 cells using Trizol (Invitrogen) and cDNA was synthesized from total RNA using the iScript cDNA synthesis kit (Bio-Rad) in a 20 μL volume. RT-qPCR was performed using a Roche LightCycler^TM^ 480II system (Roche Diagnostics, Indianapolis, IN, USA) with LightCycler^TM^ 480 SYBR Green I Master reaction mix (Roche). The sequences of primers used for RT-qPCR are displayed in Supplementary Table S1. mRNA expression levels were normalized to 18S rRNA levels.

### 2.6. Cytoplasm and Nuclear Fractionation

The cytoplasmic proteins were extracted using Cytoplasmic Extract Buffer (10 mM HEPES, 60 mM KCl, 1 mM EDTA, 0.03% NP-40, and 1 mM DTT with protease inhibitor, pH 7.6). After collection of nuclei by centrifugation at 2500 g, the nuclear proteins were extracted using Nuclear Extract Buffer (20 mM Tris.HCl, 420 mM NaCl, 1.5 mM MgCl_2_, 0.2 mM EDTA, and 25% glycerol with protease inhibitor, pH 8.0). The level of p65 was determined by western immunoblotting using the specific anti-p65 antibody (Cell Signaling Technology, Danvers, MA, USA), along with α-tubulin (Cell Signaling Technology) and histone H3 (Abcam, Cambridge, UK), which were used as internal controls for cytoplasmic and nuclear fractions, respectively.

### 2.7. Antioxidant Activity Assay

Hydroxyl Radical Antioxidant Capacity (HORAC) was measured using OxiSelect™ HORAC activity assay kit (Cell Biolabs, Inc., San Diego, CA, USA). L-ascorbic acid (1 mM) was used as control. To monitor intracellular ROS levels [22], HEI-OC1 cells were pretreated with various concentrations of DKB122 (5–100 μg/mL) and co-treated with 10 mM neomycin under the same condition in 24 h. Next, these cells were exposed to 30 μM 2,7-dichlorodihydrofluorescein diacetate (DCFH-DA; Sigma-Aldrich, St. Louis, MO, USA) according to the manufacturer’s protocol for 30 min. ROS signal was visualized by a Nikon eclipse Ti-U inverted fluorescence microscope (Nikon Instruments Inc., Tokyo, Japan). To quantify the ROS level, cells were lysed using RIPA buffer (50 mM Tris.HCl pH 8.0, 150 mM NaCl, 2 mM EDTA, 1% SDS, 1% sodium deoxycholate, and 1% NP-40), and the level of fluorescence signal was measured on 96-well white plates using a Victor X3 Multimode Plate Reader (Perkin Elmer, Waltham, MA, USA) at excitation and emission wavelengths of 480 and 530 nm, respectively. The fluorescence intensities were normalized based on cell viability.

### 2.8. Western Immunoblot

Cells were lysed using RIPA buffer (50 mM Tris.HCl pH 8.0, 150 mM NaCl, 2 mM EDTA, 1% SDS, 1% sodium deoxycholate, and 1% NP-40). The following primary antibodies were used in this study: anti-PARP (1:1000; Cell Signaling Technology), anti-LC3B (1:1000; Cell Signaling Technology), anti-p62 (1:500; Santa Cruz, TX, USA), and anti-β-actin (1:1000; Santa Cruz). Quantitative intensities were evaluated using Image Lab 5.1 (Bio-Rad, Hercules, CA, USA), followed by normalization to β-actin levels.

### 2.9. Autophagy Assay

Autophagy was monitored by western immunoblotting by measuring the formation of LC3-II using a specific antibody (Cell Signaling Technology). The formation of LC3-II was also visualized by immunofluorescence staining [22]. Briefly, cells were transfected with a plasmid expressing GFP-LC3 (Addgene Inc., Watertown, MA, USA) using the Lipofectamine™ 2000 reagent (Invitrogen) and following the manufacturer’s manual. After DKB122 (1–10 μg/mL; 8 h) or rapamycin (1 μM; 6 h; Cayman, Ann Arbor, MI, USA) treatment, cells were fixed using 4% paraformaldehyde for 10 min and then washed three times with phosphate-buffered saline for 5 min. Finally, the expression of GFP-LC3 was viewed using a confocal laser scanning microscope (Nikon, Tokyo, Japan). Cells were incubated overnight with anti-LC3B antibody (1:100, Cell Signaling Technology) at 4 °C with gentle shaking. Nuclei were stained with 1 μg/mL of Hoechst 33342 for 5 min. Autophagic flux was determined by measuring the level of LC3-II protein in the presence or absence of bafilomycin A1 (Abcam) by western blotting (Bio-Rad, Hercules, CA, USA).

### 2.10. Statistical Analysis

All statistical data were analyzed by GraphPad Prism 5.01 software (GraphPad, San Diego, CA, USA) and expressed as mean ± SD. Differences between compared groups were determined by paired t-test, where *p* < 0.05 was considered as statistically significant.

## 3. Results

### 3.1. Protection of HEI-OC1 Cells by DKB122 From Chemical-Induced Cell Death

**Figure 1:**
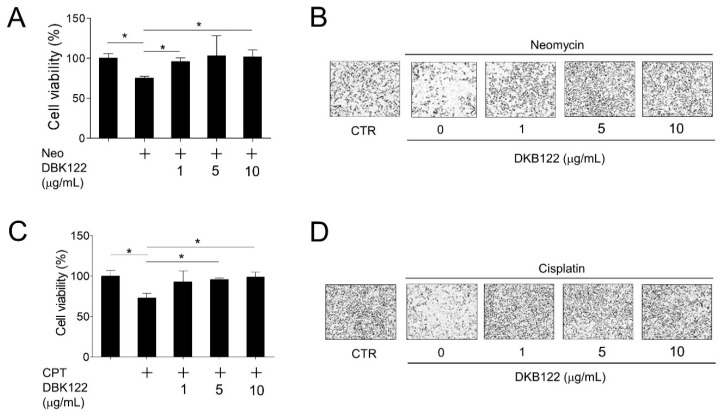
**Enhanced functional avocado oil extract** (DKB122) protects HEI-OC1 cells from chemical-induced cell death. (**A**,**B**) HEI-OC1 cells were pretreated with DKB122 (1–10 μg/mL) for 1 h, then incubated with neomycin (10 mM) for 24 h. Cell viability was determined and visualized by IncuCyte. The results are presented as mean ± SD of three independent experiments (*n* = 3); * *p* < 0.05. (**C**,**D**) HEI-OC1 cells treated with DKB122 (1–10 μg/mL) for 1 h, followed by the addition of cisplatin (CPT, 30 μM) for 24 h. Cell viability was determined and visualized by IncuCyte. The results are presented as mean ± SD of three independent experiments (*n* = 3, * *p* < 0.05).

First, the potential effects of DKB122 on hearing loss caused by chemicals were determined. Cell survival rate was measured after treatment with DKB122 in a cell-based model whereby HEI-OC1 cells were treated with neomycin to induce toxicity. Cells treated with neomycin (10 mM) for 24 h showed a decrease in viability of approximately 24.8%. By contrast, pretreatment with DKB122 (1–10 μg/mL) for 1 h improved cell viability in a concentration-dependent manner (Figure 1A,B). Similarly, pretreatment of HEI-OC1 cells with DKB122 also improved cell viability reduced by cisplatin (30 μM, 24 h), another well-known ototoxic drug [23] (Figure 1C,D). These results suggest that DKB122 may protect HEI-OC1 cells from cytotoxicity induced by representative toxic chemicals such as neomycin and cisplatin.

### 3.2. Activation Of Antioxidant Gene Expression By DKB122 In HEI-OC1 Cells

**Figure 2:**
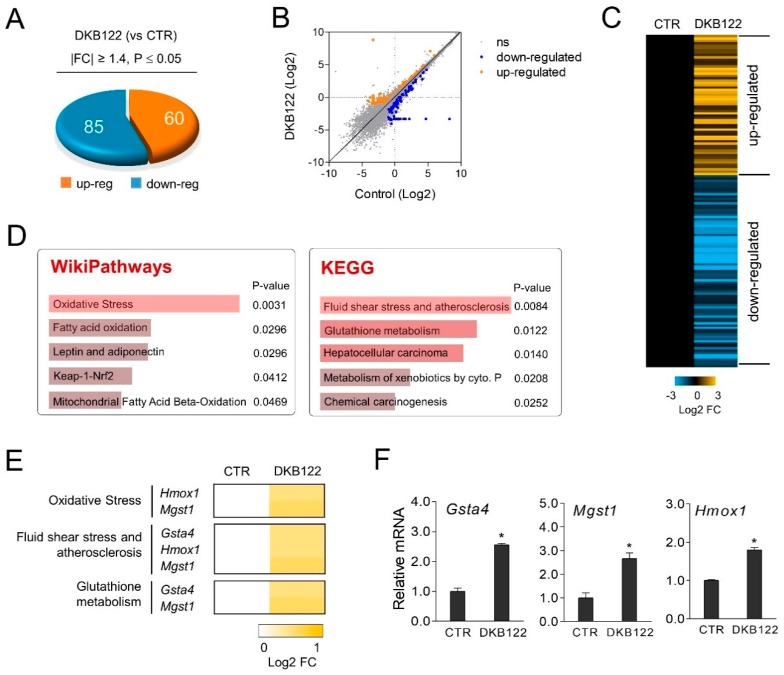
Activation of antioxidant gene expression by DKB122 in HEI-OC1 cells. (**A**,**B**) RNA-seq of DKB122 (25 μg/mL, 24 h) in HEI-OC1 cells. 85 up-regulated and 60 down-regulated genes are shown in the pie chart and scatter plot. (**C**) Heat map of gene expression changes resulting from DKB122 treatment. (**D**) Pathway analyses (WikiPathways and KEGG) of differentially expressed genes after DKB122 treatment in HEI-OC1 cells. (**E**) Heat map of genes responsible for the induction of oxidative stress, fluid shear stress, atherosclerosis, and activation of the glutathione metabolic pathway. (**F**) HEI-OC1 cells were treated with DKB122 (25 μg/mL) for 24 h prior to harvesting. Total RNA was examined by RT-qPCR. mRNA levels were normalized to *18S* rRNA. *Gsta4*, Glutathione S-Transferase Alpha 4; *Mgst1*, Microsomal Glutathione S-Transferase 1; *Hmox1*, Heme Oxygenase 1. The results are presented as mean ± SD of three independent experiments (*n* = 3, * *p* < 0.05).

RNA-seq was performed on HEI-OC1 cells after treatment with DKB122 to confirm the effective mechanism of DKB122 on ear cells. Of the 11,474 genes expressed in HEI-OC1 cells, 145 genes (85 up-regulated and 60 down-regulated) with significant expression changes (FC > 1.4, *p* < 0.05) were identified (Figure 2A–C). Next, our pathway analysis showed that genes in the oxidative stress pathway (*P* = 0.0031), the fluid shear stress and atherosclerosis pathway (*P* = 0.0084), and the glutathione metabolic (*P* = 0.0122) pathway were enriched (Figure 2D,E). Expression changes observed in the three corresponding genes (*Hmox1, Mgst1, Gsta4*) were validated by RT-qPCR (Figure 2F). All three of these genes are known to be involved in oxidative stress. Hmox1 (Heme oxygenase 1) plays an adaptive role in cellular antioxidant defense and is known to protect auditory hair cells against oxidative stress caused by cisplatin [24]. Gsta4 (glutathione transferase α4) attenuates cisplatin-induced toxicity due to detoxification in the inner ear of female mice, and depletion of Gsta4 is known to worsen hearing loss in female mice compared to male mice [25]. Mgst1 (microsomal glutathione S-transferase 1) protects against aging-related oxidative damage [26]. Indeed, it is known that overexpression of superoxide scavenging enzymes inhibits the toxicity induced by AGs in vivo [27]. These results suggest that DKB122 stimulates antioxidant genes in inner ear cells.

### 3.3. Antioxidant Effect of DKB122

**Figure 3:**
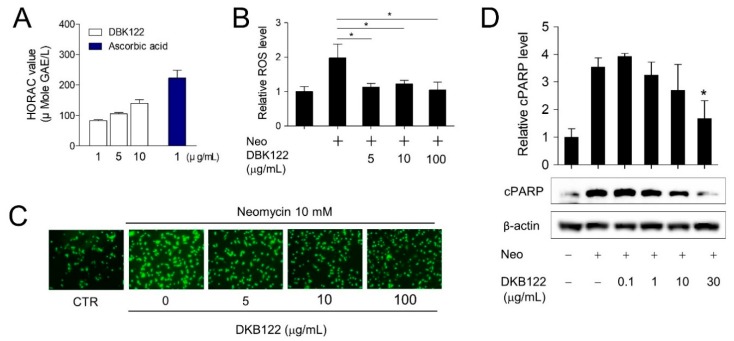
DKB122 attenuates intracellular ROS induced by neomycin and prevents apoptotic activation in HEI-OC1 cells. (**A**) HORAC assay, measuring the antioxidant activity of DKB122 (1–10 μg/mL). L-ascorbic acid (1 μg/mL) was used as the control. (**B**) HEI-OC1 cells were pretreated with DKB122 (5–100 μg/mL) for 1 h, then neomycin (10 mM) for an additional 24 h. Cells were stained with DCFH-DA (30 μM) for 30 min. ROS intensities were determined by measuring fluorescence. ROS intensities were normalized to cell viability. The results are presented as mean ± SD (*n* = 3, * *p* < 0.05). (**C**) ROS signal image obtained using fluorescence microscopy. (**D**) HEI-OC1 cells were incubated with DKB122 (0.1–30 μg/mL) 1 h prior to neomycin treatment (10 mM, 24 h). Cleaved PARP (cPARP) protein was detected using western blotting. β-actin was used as a loading control. The relative amount of cleaved PARP was evaluated using ImageJ. The results are presented as mean ± SD of four independent experiments (*n* = 4, * *p* < 0.05).

Previously, we showed that DKB122 increased the expression of antioxidant proteins in inner ear cells, and ROS generation is believed to underlie this ototoxicity [11]. Avocado oils are known to contain large amounts of unsaturated fatty acids, and their direct antioxidant effects have been reported [16,19]. Therefore, it was proposed that DKB122 can directly remove ROS generated by neomycin. Although 37.0% compared to ascorbic acid, in an in vitro antioxidant assay (HORAC assay), DKB122 showed concentration-dependent antioxidant potency (Figure 3A). To determine if DKB122 has an antioxidant effect in cells, a cell-based antioxidant assay was conducted. Exposure of HEI-OC1 cells to neomycin (10 mM) significantly increased intracellular ROS levels, and this increase was significantly reduced by DKB122 treatment (Figure 3B,C, and Supplementary Figure S1B). ROS are known to be critically involved in toxin-induced cell death [28]. Therefore, we investigated whether DKB122 inhibits neomycin-induced apoptosis. In HEI-OC1 cells, neomycin treatment caused an increase in PARP levels, an apoptosis marker, and this increase was markedly attenuated by treatment (30 μg/mL) with DKB122 (Figure 3D). Taken together, these results suggest that DKB122 inhibits the neomycin-induced cell death not only through the increased expression of antioxidant enzymes but also through the direct elimination of ROS.

### 3.4. Effects of DKB122 on the Expression of Genes Related to Noise-, Chemical-, and Age-Induced Hearing Loss

**Figure 4:**
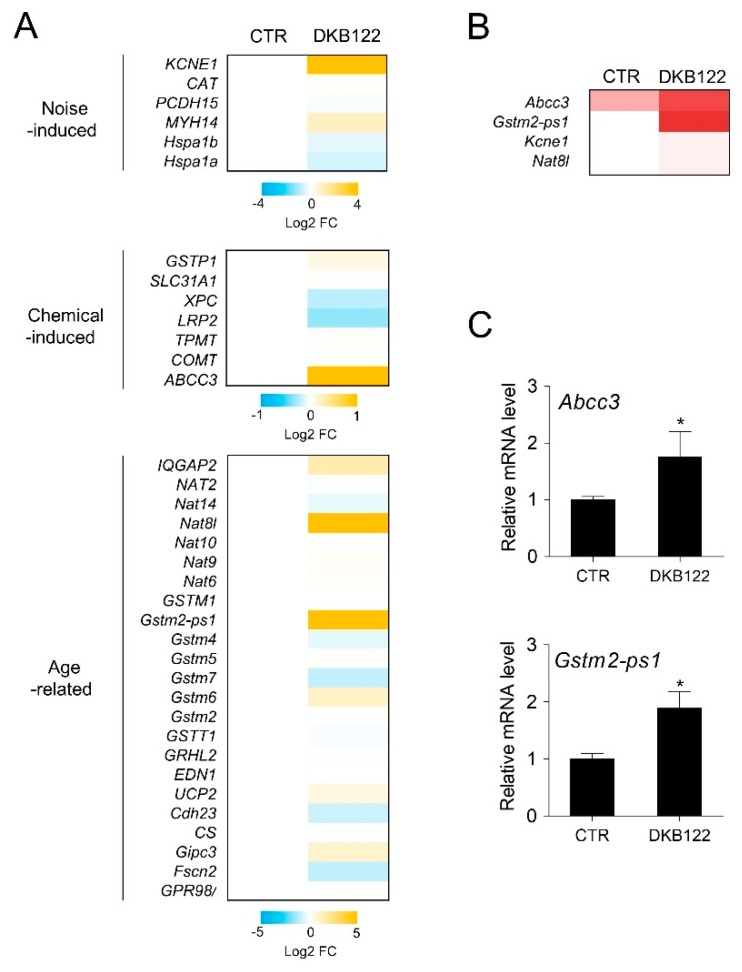
Effects of DKB122 on the expression of genes related to noise-, chemical, and age-induced hearing loss. (**A**) Heat map of genes associated with hearing loss, noise-induced, chemical-induced, and age-related hearing loss. (**B**) FPKM values of genes changed by DKB122 treatment. (**C**) mRNA levels of *Abcc3* and *Gstm2-ps1* were validated by RT-qPCR. HEI-OC1 cells were treated with DKB122 (25 μg/mL) for 24 h. Isolated RNA was quantified by RT-qPCR. mRNA levels were normalized to *18S* rRNA. *Abcc3*, ATP Binding Cassette Subfamily C Member 3; *Gstm2-ps1*, Glutathione S-transferase mu 2 (muscle), pseudogene 1. The results are presented as mean ± SD (*n* = 3, * *p* < 0.05).

In addition, we examined the effects on the expression of genes that are known to be involved in hearing loss [29,30]. The expression levels of *KCNE1*, *Abcc3*, *Nat8l*, and *Gstm2-ps1* genes were increased by DKB122 treatment (Figure 4A). However, *KCNE1* and *Nat8l* had very low expression levels in HEI-OC1 cells, which could not be validated by RT-qPCR, as opposed to *Abcc3* and *Gstm2-ps1* genes (Figure 4B,C). ABCC3 is responsible for the efflux of organic anions, heterologous biologics, and glutathione S-conjugates, and it is known that platinum chemotherapy drugs are mainly eliminated by glutathione conjugation by Abcc3 [31]. This result is consistent with previous findings that DKB122 inhibits cisplatin-induced ototoxicity and suggests one of the possible mechanisms by which endogenous cells are protected from drug-induced toxicity. This finding suggests that DKB122 has the potential to inactivate this toxic drug in HEI-OC1 cells.

### 3.5. Inhibition of the NF-kB Pathway by DKB122

**Figure 5:**
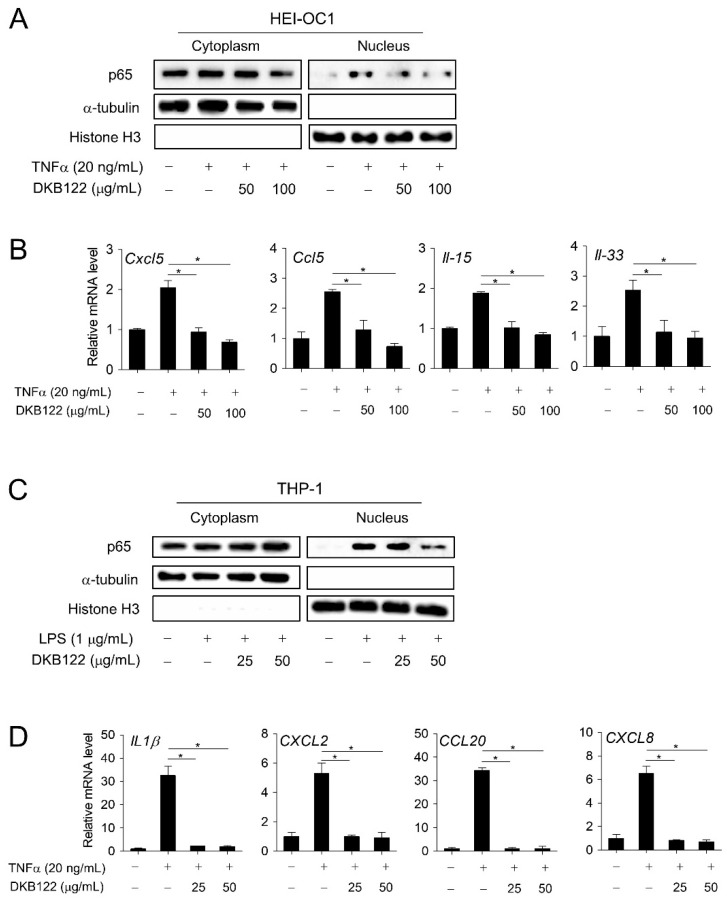
Inhibition of inflammatory gene expression by DKB122 in HEI-OC1 and THP-1 cells. (**A**) HEI-OC1 cells were pretreated with DKB122 (50–100 μg/mL) for 24 h before exposing to TNF-α (20 ng/mL, 2 h). Cells were fractionated into the cytoplasmic and nuclear fractions. p65 levels were determined by western immunoblotting using a specific anti-p65 antibody. α-Tubulin and histone H3 were used as an internal control for cytoplasmic and nuclear fractions, respectively. (**B**) HEI-OC1 cells were pretreated with DKB122 (50–100 μg/mL) for 24 h, then treated with TNF-α (20 ng/mL) for an additional 2 h. Total RNA was harvested and examined by RT-qPCR. mRNA levels of each gene were normalized to 18S rRNA. The results are presented as mean ± SD of three independent experiments (*n* = 3, * *p* < 0.05). (**C**) THP-1 cells were pretreated with DKB122 (25–50 μg/mL) for 1 h, then LPS (1 μg/mL, 30 min). p65 levels in the cytoplasmic and nuclear fractions were determined by western immunoblotting as in Figure 4A. (**D**) THP-1 cells were pretreated with DKB122 (25–50 μg/mL) for 1 h and then with TNF-α (20 ng/mL) for an additional 6 h. Total RNA was harvested and examined by RT-qPCR. mRNA levels of each gene were determined as in Figure 4B. The results are presented as mean ± SD of three independent experiments (*n* = 3, * *p* < 0.05).

In an in vivo model, coordination of endotoxin lipopolysaccharides and AGs increases AG uptake in the cochlea, resulting in more severe cochlear inflammation [10]. Earlier RNA-seq results showed that DKB122 inhibits the expression of genes encoding chemokines (*Ccl5, Cxcl5, Cxcl10*, and *Cxcl12*) and interleukins (*Il-15*) in HEI-OC1 cells (Supplementary Figure S2). Correspondingly, DKB122 inhibited the nuclear translocation of p65 induced by TNFα (20 ng/mL) in HEI-OC1 cells (Figure 5A). Consistent with this, DKB122 treatment significantly inhibited the expression of inflammatory genes induced by TNF-α (20 ng/mL) determined by RT-qPCR (Figure 5B and Supplementary Figure S1C).

Cytokines are initially induced in response to inflammatory stimuli, which then attract and stimulate immune cells. It is also known that immune cells reside in the ear, where they function as a defense against active external pathogens [32]. Macrophages can migrate within the uninfected inner ear in response to noise, AGs, and aging. This causes cochlear damage in a variety of ways [33]. Recent studies show high expression of CC- and CXC-type chemokines (*Cxcl10, Cxcl12, Ccl5*, etc.) in response to inflammatory activation induced by 3-nitropropionic acid on the side wall of mouse cochlea [34]. Therefore, we investigated the effect of DKB122 on the inflammatory pathway in human monocytes. DKB122 inhibited the nuclear translocation of p65 induced by LPS (1 μg/mL) in THP-1 cells (Figure 5C). Expression levels of *IL-1β* and other cytokines (*CXCL2, CXCL8, CCL20*) increased by TNFα treatment were also significantly inhibited by treatment with DKB122 (Figure 5D, Supplementary Figure S1D). Taken together, these data suggest an anti-inflammatory mechanism by which DKB122 exhibits its protective effects in HEI-OC1 cells.

### 3.6. Activation of Autophagic Flux by DKB122

**Figure 6:**
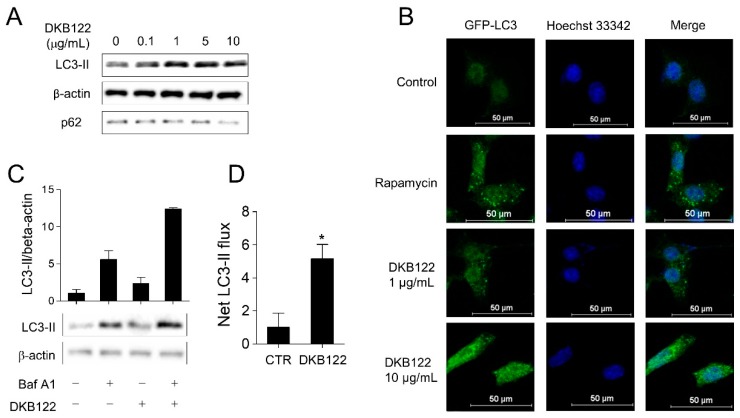
Activation of autophagic flux by DKB122. (**A**) HEI-OC1 cells were incubated with DKB122 (0.1–10 μg/mL) for 24 h, then harvested. LC3-II and p62 protein levels were detected using western immunoblotting. β-Actin was used as a loading control. (**B**) After transfection with a plasmid expressing GFP-LC3, HEI-OC1 cells were treated with DKB122 (1–10 μg/mL) for 8 h. Rapamycin (1 μM, 6 h) was used as a positive control. Intracellular GFP-LC3 puncta (green) were tracked using confocal microscopy. Nuclei were stained with Hoechst 33342 (blue). (**C**) HEI-OC1 cells were exposed to DKB122 (5 μg/mL, 8 h) and/or Bafilomycin A1 (1.5 nM, 4 h). Then, the LC3-II level was examined using western immunoblotting. (**D**) The increase in autophagic flux by DKB122 was calculated in comparison to that of the untreated group. The results are presented as mean ± SD (*n* = 3, * *p* < 0.05).

A variety of fatty acids are found in avocado oils, the four highest being oleic acid, palmitic acid, linoleic acid, and palmitoleic acid [35,36]. Oleic acid, linoleic acid, and palmitic acid are known to activate autophagy through mTOR-dependent or -independent pathways [37,38,39], and autophagy can improve inner ear cell survival by reducing oxidative stress levels in noise-induced hearing loss [40]. Therefore, we hypothesized that DKB122 can regulate autophagy to provide otoprotection. To test this, we first treated HEI-OC1 cells with a range of doses of DKB122 (0.1–30 μg/mL). Accumulation of the autophagy marker LC3-II protein occurred in a dose-dependent manner (Figure 6A). P62 (Sqstm1/sequestome 1), which binds to LC3, is selectively incorporated into the autophagosome, resulting in a decrease in intracellular protein levels of p62 with increasing autophagic flux. Treatment with DKB122 resulted in a decrease in p62 protein in a dose-dependent manner, suggesting that DKB122 increased autophagy in HEI-OC1 cells (Figure 6A). Next, we treated HEI-OC1 cells overexpressing GFP-LC3 with DKB122 (1–10 μg/mL), which resulted in an increase in the number of intracellular LC3-II puncta in comparison to the control group (Figure 6B). Rapamycin was used as a positive control for autophagy activation. Finally, we measured LC3-II protein turnover in the presence or absence of the autophagy inhibitor Bafilomycin A1 (BafA1) in order to assess autophagic flux [41]. As shown in Figure 6C, the accumulation of LC3-II cells by DKB122 was significantly increased, while the net flux of LC3-II was increased by 5.16 times, compared to the control group (Figure 6D).

Taken together, these results suggest that DKB122 induces autophagy in HEI-OC1 cells. Autophagy is known to inhibit apoptosis and protect hair cells after AG exposure [42]. Thus, our results validate that DKB122 inhibits neomycin-induced apoptosis via autophagy activation.

## 4. Discussion

Hearing loss is a general sensory deficiency characterized by a decrease in hearing sensitivity. Hearing impairment can be caused by noise, drugs, aging, and infections. The regenerative ability of mammalian cochlear tissue is so limited that once damaged, hair cells are incapable of repair, which means permanent loss of function [43]. Therefore, the discovery of otoprotectants is important for treating hearing loss in mammals. Here, we investigated the mechanism by which avocado oil extract (DKB122) exerts its protective effects on drug-induced hearing impairment.

To illustrate the mode of intracellular action, we performed RNA-seq to identify changes in gene expression in response to DKB122. As a result, it was confirmed that pathways related to oxidative stress defense and glutathione metabolism are activated by DKB122. Even in the HEI-OC1 cell-based model, we found that DKB122 clearly reduced neomycin-stimulated ROS levels. It was also confirmed that DKB122 itself was capable of direct ROS removal. ROS have been reported to play an important role in AG- or cisplatin-induced hair cell death [23,44,45]. In addition, excessive amounts of intracellular ROS are believed to be key players in the development of both age-related and noise-induced hearing disorders [46]. Therefore, ROS neutralization has been studied extensively as a potential therapeutic target in the treatment of hearing loss. This study provides cell-based evidence that DKB122 can treat hearing impairment by weakening intracellular ROS levels.

Inflammation caused by bacterial infection has been reported to exacerbate AG-enhanced toxicity in the cochlea due to the upregulation of the AG-permeable cation channel TRPV1 [47]. In addition, pre-inflammatory cytokines account for this toxicity enhanced by cisplatin, another toxic reagent in cell-based and animal studies [48,49], thus highlighting the importance of inflammation in hearing disorders originating from various causes, such as noise and aging [50]. In this study, we demonstrated that DKB122 inhibits the expression of chemokine- and interleukin-related genes by regulating the nuclear translocation of NF-κB in auditory blast cells. Anti-inflammatory agents have been reported to alleviate gentamicin-induced hearing loss in vivo and in clinical studies [28]. Avocado oil is very rich in unsaturated fatty acids, and oleic acid, in particular, comprises the highest proportion of total fatty acids in avocado oil [35]. Oleic acid has been reported to upregulate anti-inflammatory cytokines as well as down-regulate pro-inflammatory cytokines in septic mice [51]. It also effectively reverses TNF-α induced insulin inhibition in vivo, which points to the possibility of its use in the alleviation of inflammatory symptoms of type II diabetes [52]. The anti-inflammatory action of DKB122 appears to favor the treatment of hearing loss caused by a variety of factors.

Most recent work has shown that autophagy contributes to the morphology and function of auditory blast cells [53]. Blocking autophagy flux by AG results in the accumulation of autophagosome bodies, resulting in ear toxicity. By contrast, enhancement of autophagic flux is believed to improve AG-induced [54] and cisplatin-induced [55] auditory cell death. The antioxidant aspect of autophagy also acts as an important defense mechanism for other types of acquired hearing loss, such as noise-induced and age-related hearing loss. In particular, AG is distributed to blast cells via non-endodermal and intracellular pathways and accumulates in lysosomes from the extracellular space [56]. Lysosomes are the sites of degradation of molecules trapped in autophagosomes, and AGs disrupt the function of lysosomes to inhibit the autophagic flux [57], which leads to further toxicity and increased hair cell death [56]. These findings are consistent with the antioxidant role of autophagy in protecting hair cells from neomycin-induced cell death. In addition, a positive shift in autophagy activity by DBK122 carries a powerful strategy for controlling the side effects of other toxic drugs such as gentamicin and cisplatin, both in vitro and in vivo. Thus, autophagy is of great importance for protecting ear cells against AG-induced damage.

In summary, our data demonstrate that DKB122 can protect auditory hair cells from neomycin-induced damage through direct or indirect antioxidant pathways, inhibition of inflammatory gene expression, and autophagic activation.

## Supplementary Materials

The following are available online at https://www.mdpi.com/2072-6643/12/4/947/s1, Figure S1: Effect of DKB122 alone treatment on HEI-OC1 cells. Figure S2: DKB122 suppresses inflammatory gene expression in normal ear cells. Table S1: Primer sequences for RT-qPCR.
