# Supplementary material for: Protective Mechanisms of Avocado Oil Extract Against Ototoxicity
Source: Nutrients. 2020 Mar 29;12(4):947. doi: 10.3390/nu12040947 (PMC7230542; doi:10.3390/nu12040947)
Supplement: Supplementary file 1 [file nutrients-12-00947-s001.pdf]

Supplementary Information

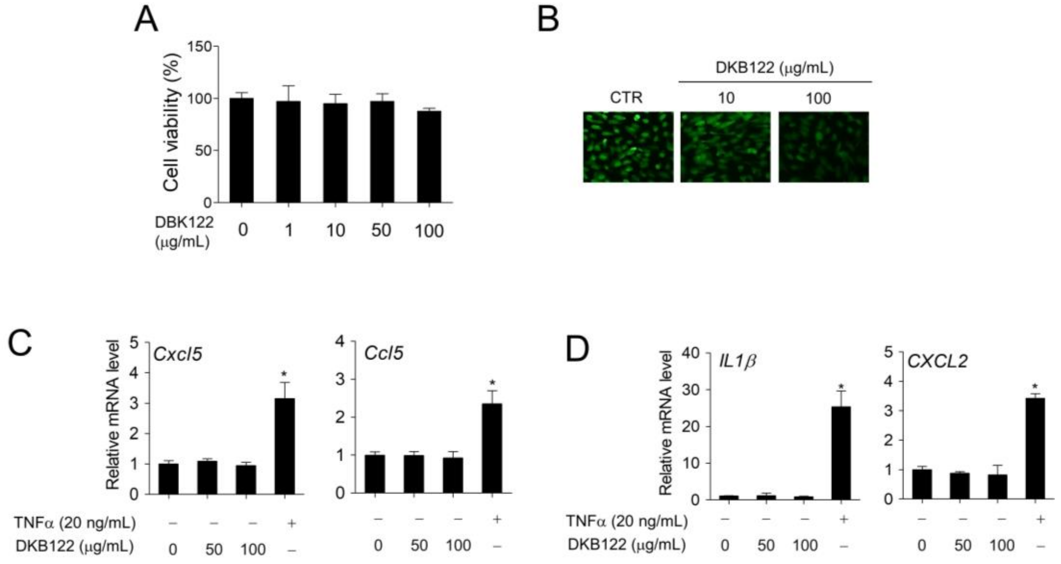

Supplementary Figure S1. Effect of DKB122 alone treatment on HEI-OC1 cells. (**A**) HEI-OC1 cells were treated with DKB122 (1–100 μg/mL) for 24 h. Cell viability was determined by IncuCyte. (**B**) ROS signal images obtained using fluorescence microscopy from HEI-OC1 cells treated with DBK122. (**C,D**) HEI-OC1 cells were treated with DKB122 (50–100 μg/mL) for 24 h or TNF-*α* (20 ng/mL) for 2 h. The expression levels of several genes related to chemokines and interleukins were validated by RT-qPCR. mRNA levels were normalized to *18S* rRNA. (n = 3, * P < 0.05).

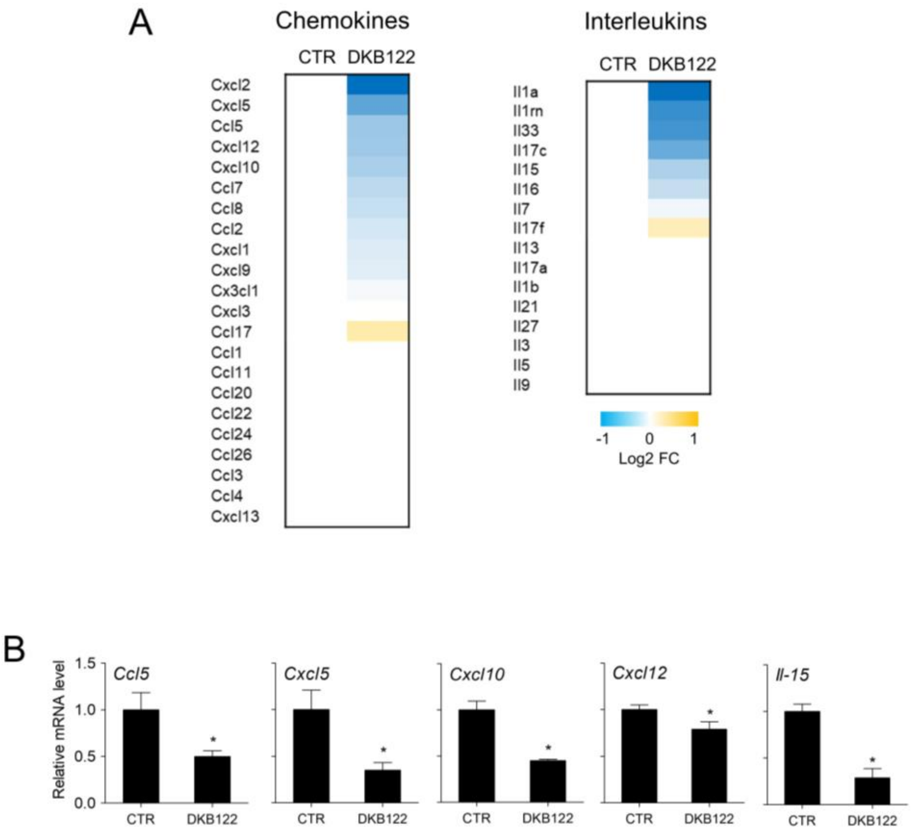

Supplementary Figure S2. DKB122 suppresses inflammatory gene expression in normal ear cells. (**A**) Heat map generated from RNA-seq using the genes related to chemokines and interleukins. (**B**) The expression levels of several genes related to chemokines and interleukins were validated by RT-qPCR. HEI-OC1 cells were incubated with DKB122 (25 µg/mL) for 24 h before harvested. Total RNA was examined by RT-qPCR. mRNA levels were normalized to 18S rRNA. The results are presented as mean ± SD of three independent experiments ($n = 3$, * $P < 0.05$).

Supplementary Table S1. Primer sequences for RT-qPCR.

**Name**	**Forward**	**Reverse**	**Species**
IL1β	CCACCTCCAGGGACAGGATA	AACACGCAGGACAGGTACAG	human
CCL20	TGTCAGTGCTGCTACTCC	CCAAGACAGCAGTCAAAGTTGC	human
CXCL2	ATCGCCCATGGTTAAGAAAA	CTTCAGGAACAGCCACCAAT	human
CXCL8	TGAATTACGGAATAATGAGTTAGAAC	TCAACCAGCAAGAAATTACTAAT	human
18S	GAGGATGAGGTGGAACGTGT	TCTTCAGTCGCTCCAGGTCT	human
Hmox1	ccccagatcagcactagctc	atggcataaattcccactgc	mouse
Mgst1	agcccacctgaatgatcttg	tgaagtgcatgagggctgta	mouse
Gsta4	cccttggttgaaatcgatgg	gaggatggccctggtctgt	mouse
Gstm2-ps1	tcccaaacctgagggactt	caggtgttgcgatgtagcag	mouse
Abcc3	tcccacttttcggagacagtaac	actgaggaccttgaagtcttgga	mouse
Cxcl5	cactcgcagtggaaagaacg	cgtgggtggagagaatcagc	mouse
Ccl5	agatctctgcagctgccctca	ggagcacttgctgctggtgtag	mouse
Il-15	catccatctcgtgctacttgtgtt	catctatccagttggcctctgttt	mouse
Il-33	gatgggaagaagctgatggtg	ttgtgaaggacgaagaaggc	mouse
18s	gtaacccgttgaaccccatt	ccatccaatcggtagtagcg	mouse
